# Breeding success but not mate choice is phenotype- and context-dependent in a color polymorphic raptor

**DOI:** 10.1093/beheco/arz013

**Published:** 2019-02-09

**Authors:** Laura Gangoso, Jordi Figuerola

**Affiliations:** 1 Institute for Biodiversity and Ecosystem Dynamics, University of Amsterdam, Amsterdam, The Netherlands; 2 Department of Wetland Ecology, Estación Biológica de Doñana, CSIC, Sevilla, Spain

**Keywords:** breeding output, color polymorphism, Eleonora’s falcon, inbreeding, mate choice, natal dispersal

## Abstract

Morph-specific mate choice has been proposed as one of the evolutionary mechanisms that contribute to the maintenance of variation in color polymorphic systems. Coloration usually covaries with other phenotypic traits affecting life history and thus is often used as a criterion for mate choice. Here, we assess whether mating patterns, natal dispersal, and breeding output are phenotype-dependent in the color polymorphic Eleonora’s falcon. We used a long-term dataset of 946 individually ringed adult falcons that included 109 individuals monitored from birth up to recruitment into the breeding population. Overall, patterns of mate choice with regard to coloration were neither assortative nor disassortative. Natal dispersal distance was greater in females but was not associated with coloration. Breeding success was both morph-dependent and context-dependent. Although clutch size was similar in differently colored pairs, differences arose in the number of chicks that fledge. In some years, dark males raised more offspring, regardless of female color morph. Differences in the breeding tactics between male morphs could be associated with intraspecific predation and may thus contribute to the observed differences in breeding output, especially when food availability is low. This suggests that mating patterns may interact with other factors and give rise to the observed higher breeding output of dark males only under certain environmental conditions.

## Teaser

In color polymorphic species, individuals can prefer mates of the same or different color, change preference according to socio-environmental conditions, or even show no preference. Coloration can advertise many individual qualities, including kinship. Eleonora’s falcons morphs do not mate assortatively or disassortatively, although two incestuous pairs were of the same dark-morph. Dark males raised more offspring than pale ones, but not all years, suggesting that mating interactions in turn interact with environmental factors.

## INTRODUCTION

A major challenge in conservation and evolutionary biology is that of unraveling the mechanisms and evolutionary processes involved in the maintenance of discrete genetic polymorphisms in spatially or temporally variable environments (Levene 1953; Gillespie 1973). Intraspecific heritable color variation is a conspicuous widespread case of genetic polymorphism that is likely maintained by balancing selection (either via over-dominance or negative frequency-dependent selection) or correlational selection (i.e., selection for combinations of traits) (Svensson 2017). In this regard, mating patterns can play a decisive role and may be affected by the color of morphs due to these selective processes (see review in Wellenreuther et al. 2014). In vertebrates, color polymorphism has been shown to be related to a large array of individual qualities, life history, and behavioral traits (Roulin et al. 2001; Ducrest et al. 2008; Calsbeek et al. 2010; Galeotti et al. 2013), which raises the possibility that individuals assess this condition-independent trait as a criterion for mate choice. Indeed, the frequent occurrence of nonrandom mating in terms of coloration supports the hypothesis that color is used as an important cue during reproduction (Roulin 2004; Wellenreuther et al. 2014).

In polymorphic systems, mate choice may be context- or phenotype-dependent (see review in Roulin and Bize 2007). Mate choice will be context-dependent if a particular morph is temporally better adapted to certain environmental or social conditions. In this case, all individuals will show the same preference for a particular morph, a temporal choice that may shift or be relaxed over time as environmental conditions and/or relative morph frequencies change. For example, the mating preferences of the melanin-based color dimorphic ladybird beetle females (*Harmonia asyridis*) change from nonmelanic males in spring to melanic males in summer (Osawa and Nishida 1992). Context-dependent mate choice can also occur when alternative morphs exploit different niches or have different breeding strategies that are asymmetrically advantageous under different environments. For instance, female pygmy swordtails (*Xiphophorus pygmaeus*) only prefer blue males over dominant and more aggressive gold males in certain populations where the predation risk is low (Kingston et al. 2003). However, mating may also be dependent on the own-phenotype or that of relatives. In this latter case, between-individual variation in mate-choice decisions can be sensitive to early imprinting, that is, both sexes are more likely to mate with an individual that resembles a parent or siblings (Price 1998; ten Cate and Vos 1999). In any case, under phenotype-dependent mate choice, patterns of either positive or negative assortative mating with regard to coloration are expected to occur.

Coloration may not only affect mating patterns but also other life-history traits such as dispersal strategies. For example, van den Brink et al. (2012) found that darker pheomelanic barn owls (*Tyto alba*) dispersed further from birth to breeding sites than paler conspecifics and suggest that the relationship between natal dispersal and coloration is genetically inherited. Natal dispersal can have diverse adaptive functions and one of its major advantages is thought to be the avoidance of inbreeding (Dobson and Jones 1985), a hypothesis supported by the frequency of sex biases in dispersal patterns that probably reduce co-ancestry between potential mates (Perrin and Mazalov 1999). Color-specific dispersal could be understood in this context if, for instance, one morph is more sensitive to inbreeding than the other (van den Brink et al. 2012), although evidence for this possibility is still lacking.

Assuming that assortative mating has an adaptive function, it has been proposed that positive assortative mating may be related to genetic compatibility or benefits from offspring homozygous at genes coding for coloration or at closely linked genes (Roulin and Bize 2007). If assortative mating were meaningful, homotypic pairs would have greater reproductive success than heterotypic ones (e.g., Saino and Villa 1992). Nonetheless, if this strategy was consistent over time, it could lead to behavioral reproductive isolation, thereby hampering the maintenance of genetic variation (Richards-Zawaki et al. 2012). By contrast, negative-assortative mating may increase fitness through heterozygous offspring or by avoiding inbreeding, but also by maximizing the capacity of the pair to, for example, successfully forage under differing environmental conditions (e.g., Tate et al. 2017). However, the reliability of these mechanisms is far from being generalized and little is known about the benefits of adopting one or another mate-choice rule (see review in Roulin and Bize 2007). In addition, it is important to note that, in contrast to condition-dependent traits, morph-specific mate choice is not necessarily unidirectional (Roulin and Bize 2007). Individual preference may vary over time since color polymorphism may signal quality and also adaptation to specific but varying environmental conditions.

Our aim was to 1) test the relationship between plumage coloration and mate choice in a wild population of a color polymorphic long-distance migratory raptor; 2) compare the natal dispersal of both color morphs; and 3) analyze the relationship between individual and pair morph characteristics and breeding success. We addressed these objectives using a long-term dataset on individually ringed Eleonora’s falcons (*Falco eleonorae*). This species has a melanin-based discrete polymorphism due to variation at the *Mc1r* gene and morphs are inherited in a simple Mendelian fashion (Gangoso et al. 2011). Morph frequencies in our study population (30% dark vs. 70% pale) are apparently stable over time (Gangoso et al. 2015a). Although Eleonora’s falcons of different color morphs have been found to differ in several physiological and behavioral traits (Gangoso et al. 2011, 2015a, 2015b; Galván et al. 2010), as yet we are unsure whether the information that morph type conveys is used as a criterion in mate choice.

## MATERIAL AND METHODS

### Field procedure

This study was carried out on Alegranza (1050 ha, 289 m a.s.l.), the northernmost island of the Canary Archipelago. Eleonora’s Falcons feed on migratory birds that are pushed off course from their migratory routes by easterly winds. Food resources are found outside the colony and their availability depends heavily on external abiotic factors (Gangoso et al. 2013; Viana et al. 2016). There are no mammal predators on the island and the habitat is virtually homogeneous in terms of the environmental variables determining landscape structure (Gangoso et al. 2015b). This suggests that any differences in breeding output between breeding pairs will be chiefly associated with individual quality and hunting abilities.

The Eleonora’s falcon population on Alegranza consists on average of 127 breeding pairs (range = 110–132 pairs), which were intensively monitored in July–October 2007–2017. Every year and for each nest-site, we recorded the morph of both parents using a spotting scope (*N* = 1008 nesting events). The reliability of our visual morph scoring was confirmed by molecular analyses (Gangoso et al. 2011). The dark allele is dominant over the pale one and pale individuals are homozygous; by contrast, dark birds are either heterozygous or homozygous. However, the frequency of dark homozygous falcons is extremely low (0.7% in nestlings and less than 2% in adults (Gangoso et al. 2015a) and so we grouped all dark heterozygous and homozygous (*N* = 3 individuals during the 11-year study period) individuals together simply as dark morphs. Nests were visited on at least 2 occasions; first, to quantify clutch size and record the geographic position on a GPS and, second, to determine the final number of fledglings. During the study period, a total of 1566 Eleonora’s falcons were banded with conventional metal rings. Of these, 1186 individuals were also banded with colored plastic rings that allow for individual identification at great distances. In addition, 109 individuals (pale morph: 47 females and 43 males; dark morph: 5 females and 14 males) in the breeding population were monitored from birth to recruitment in successive years. Therefore, the color morphs of the band bearer, its mate, and both parents are known for this subset of 109 individuals (Supplementary Table S1).

### Statistical analyses

For 62 nests, the color morph of only one of the parents was known, which reduced the initial sample size to 946 nests (Table 1). The occurrence of negative- or positive-assortative mating was assessed by performing a mixed-effects logistic regression (MLR) model using the package *lme4* (Bates et al. 2015) for R software 3.4.3 (R Core Team 2017). We used the color morph of the male as the dependent variable, the color of the female as a fixed factor, and year as a random term. We also analyzed patterns across years using Fisher’s exact test, which gave qualitatively identical results (not shown).

**Table 1:** Morph composition of the Eleonora’s falcon breeding pairs during the study period 2007–2017

Year	Pale-Pale	Pale-Dark	Dark-Pale	Dark-Dark
2007 (101)	63	14	22	2
2008 (85)	57	15	10	3
2009 (103)	67	18	15	3
2010 (76)	48	12	14	2
2011 (82)	55	10	15	2
2012 (96)	69	12	13	2
2013 (79)	49	11	15	4
2014 (65)	45	8	9	3
2015 (101)	65	14	19	3
2016 (62)	39	8	11	4
2017 (96)	70	8	15	3

The first row shows the different male–female color pairs composition. The number of breeding pairs monitored is shown in brackets next to each year.

To assess patterns of mating within the subset of recruited individuals (*N* = 109), we fitted a LR model including the color morph of the recruited individual and its sex as fixed factors, and the color morph of the mate as the dependent variable. During the study period, we performed a cross-fostering experiment in which 32 complete clutches were swapped between differently colored pairs (Gangoso et al. 2015a). Thirteen of these cross-fostered individuals were recruited as adult breeders some years later. Therefore, we also included the color morph of both biological and foster parents as independent variables in the model. For the individuals that were not cross-fostered (*N* = 96), the color morph of biological and foster parents was the same. Some individuals (*N* = 44) recruited as a pair in the same nest-site. We could not include the nest ID as a random term due to convergence problems. Therefore, to avoid pseudoreplication issues, we randomly choose one individual from these 22 pairs, which reduced the sample size to 87 individuals, and run the LR analysis. We also run a similar analysis by using the previously discarded individuals instead of the initially chosen ones, which produced a new dataset of 87 individuals.

For the recruited individuals, we also used the geographic coordinates of the two nest sites to calculate the linear dispersal distances in meters from the natal nest to the nest-site at which the bird was recruited as a breeder. We assessed the relationship between color morph and natal dispersal distance using a generalized linear mixed model (GLMM). Dispersal distance was log-transformed to attain normality and was included as the response variable in the model, with the sex and color morph of the recruited individual and their interaction included as fixed factors. Age of recruitment (mean = 4 years, range = 2–8 years) was included as a random term. We did not include morphological traits measured as fledglings because these do not correspond to the body condition or size of the same individual several years later. Eleonora’s falcons perform a migration of about 10 000 km twice a year (Kassara et al. 2017) and variation in morphological traits such as wing length does not seem to limit natal dispersal distances.

Since nests differ in the number of eggs laid and, consequently, in the maximum number of chicks that can fledge, we compared breeding output using 2 parameters: 1) clutch size, estimated as the number of eggs present during the first visit to the nest, and 2) productivity, estimated as the number of chicks present during the second visit or detected using a spotting scope when nestlings were ≥35 days old. For these analyses, we considered only those pairs that initiated reproduction, that is, that laid eggs. However, clutch size was not recorded in all nests, which reduced the initial sample size to 740 breeding attempts. In 2014, although Eleonora’s falcons initiated reproduction, and consequently, we recorded clutch size, no nestlings fledged that year. Most of them died at very short age due to a generalized and prolonged starvation period associated with bad weather conditions (Viana et al. 2016). We therefore excluded 2014 for the analysis of productivity, which further reduced the sample size to 681 breeding attempts spread over 10 years.

Differences in clutch size between differently colored individuals were tested using cumulative link mixed models (CLMM) fitted with the Laplace approximation, and the *clmm2* function in the *ordinal* package (Christensen 2018). In the CLMM, we used the ordered response variable clutch size (4 > 3 > 2 > 1) and included the color morph of both parents plus their interaction as fixed factors, and year as a random term.

We performed a similar CLMM in which productivity was included as the ordered response variable (4 > 3 > 2 > 1 > 0). Fixed and random factors were included as above but clutch size was also included as a covariate. Fixed effects were tested using likelihood ratio tests, after which we performed post hoc level multiple comparisons using the function *lsmeans* with the Tuckey adjustment in the package *emmeans* (Lenth 2018).

Given that productivity varied greatly during the study years (likelihood ratio test, LR stat = 117.65, df = 9, *P* < 0.001) and in order to assess patterns over time, we also performed a separate CLM for each year, in which productivity was the dependent variable and the color morph of the male the only fixed factor.

## RESULTS

We found no evidence of positive or negative assortative mating with regard to color morph in all study years (female morph χ^2^ = 0.06, *P* = 0.80). For the subsample of recruits, targeted individuals did not mate more frequently with individuals of the same color morph (model considering the initially randomly chosen individuals from those that recruited as a pair in the same nest-site: χ^2^ = 0.91, df = 1, *P* = 0.34; model considering the remaining previously discarded individuals: χ^2^ = 0.48, df = 1, *P* = 0.49). Mate choice was unrelated to the sex of the recruit or the color morph of the biological and foster parents (in all cases, *P* > 0.2, Supplementary Table S2). Females dispersed over greater distances than males (mean ± SE = 1824.39 ± 246.52 m and 910.40 ± 120.90 m, respectively, χ^2^= 17.72, df = 1, *P* < 0.001). Natal dispersal distances did not depend on the color morph of the recruit (χ^2^= 0.56, df = 1, *P* = 0.45) or its interaction with sex (χ^2^= 0.10, df = 1, *P* = 0.75).

**Figure 1:**
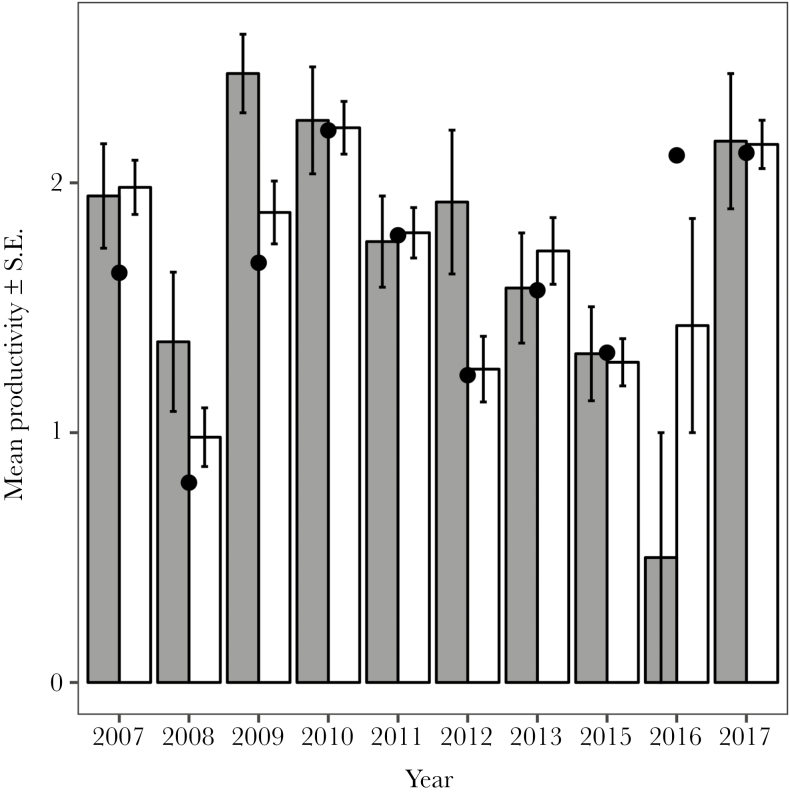
Mean productivity (number of fledglings) ± standard error of breeding pairs in dark (dark gray) or pale (light gray) male Eleonora’s falcons (2014 is not shown because no chicks fledged that year). Black dots represent data on annual mean productivity (number of fledglings/monitored breeding pair) of the entire population (*N* = 998, including breeding pairs where the color morph of only one of the parents was known).

Clutch size did not depend on either the color morph of adults or their interaction (in all cases, *P* > 0.5; Supplementary Table S3). Productivity was positively related to clutch size (estimate: 1.70 ± 0.14 SE, *z*-value = 12.01, *P* < 0.001, likelihood ratio test LR stat = 165.25, df = 1, *P* < 0.001). Dark males raised more fledglings than pale ones (estimate pale male: −0.36 ± 0.18 SE, *z*-value = −2.01, *P* < 0.04, lsmeans contrast estimate = 0.36 ± 0.18 SE, LR stat = 4.04, df = 1, *P* = 0.04, Figure 1; Supplementary Figure A1 shows the fitted probabilities of the model). The color morph of the female (LR stat = 0.001, df = 1, *P* = 0.99) and its interaction with the color morph of the male (LR stat = 1.37, df = 1, *P* = 0.24) did not significantly affect variation in productivity. However, dark males raised significantly more fledglings in only 2 of the study years (2009: estimate =1.06 ± 0.53 SE, *z*-value = 2.01, *P* = 0.04 and 2012: estimate = 1.20 ± 0.58 SE, *z*-value = 2.06, *P* = 0.04, Figure 1), whereas nonsignificant differences occurred in the other years.

## DISCUSSION

Morph-specific mate choice has been proposed as one of the evolutionary mechanisms that promotes variation and hence contributes to the maintenance of color polymorphism in natural populations (Roulin 2004; Pryke and Griffith 2007; Pérez i de Lanuza et al. 2012) and can even lead to sympatric or allopatric divergence due to reproductive isolation (Puebla et al. 2007; Reynolds and Fitzpatrick 2007; Elmer et al. 2009). Patterns of positive assortative and, to a lesser extent, disassortative mating have been widely reported in polymorphic bird species (Roulin 2004), suggesting the prominent role of nonrandom mate choice in generating color-based sexual selection (Wellenreuther et al. 2014). Patterns of disassortative mating have been reported in other Eleonora’s falcon populations (Walter 1979), but in our study we found no evidence for assortative or disassortative mating with regard to coloration over time. This result is further supported by the fact that widowed individuals mate with conspecifics of different color morphs in successive years. In males, 50% of pale widowers (*N* = 2) changed the color of their new mates from pale to dark, whereas 100% of dark widowers (*N* = 2) did not change the color morph when remating. In females, 100% of pale widows (*N* = 2) shifted from pale to dark and vice versa in successive pairings, whereas 50% of dark widows (*N* = 2) changed the color of its mate from dark to pale. The fact that alleles responsible for the expression of color polymorphism conform to the Hardy–Weinberg equilibrium (Gangoso et al. 2015a, 2015b) also supports that mating is not assortative or disassortative in this population. Extra-pair paternity could have important repercussions for inferring the influence of parental genotype. Although we cannot completely rule out this possibility, multilocus DNA fingerprinting analyses performed by Wink and Ristow (2000) showed that extra-pair paternity was almost inexistent in this species. Sexual preferences were not influenced by learned phenotypes. Although sexual imprinting early in development is a widespread phenomenon in birds (ten Cate and Vos 1999) and has been reported in some color polymorphic species (e.g., Cooke et al. 1976; Roulin 1999), Eleonora’s falcons do not choose partners resembling the color morph of their biological or foster parents. Likewise, Pryke (2010) showed that cross-fostered Gouldian finches (*Erythrura gouldiae*) were not imprinted by the phenotype of their conspecific or heterospecific foster parents, thereby supporting the idea that mating preferences in Gouldian finches are not learned but are rather genetically determined, although in this case finches mated assortatively. Our results would therefore suggest that coloration in Eleonora’s falcons is selectively neutral with respect to mate choice.

Within the subset of individuals monitored from birth to recruitment, we found similar lack of evidence for assortative or disassortative mating. However, we detected the occurrence of 2 incestuous pairings between dark full siblings: 2 dark heterozygous and 2 dark homozygous siblings. Despite the fact that the frequency of the pale morph is higher than that of the dark morph in our study population (70% vs. 30%, respectively), no case of pairing between broodmates was ever detected in pale falcons in the subset of recruited individuals (frequency: 21% in the dark morph and 0% in the pale morph). Given the Mendelian inheritance of this trait, color morph may thus be a poor predictor of parental genotype and the degree of relatedness of conspecifics; however, the probabilities are far higher in the dark morph. Dark homozygous falcons can only result from dark-dark pairs (3.28% of total pairs, *N* = 946) and, given the low frequency of the dark morph (28% of dark heterozygous falcons, which would produce dark homozygous offspring at a probability of 25%), this occurrence is necessarily rare and is favored by inbreeding. The probability of a dark homozygous male paired with a dark homozygous female is only 0.04% (Ristow et al. 2000), but the probability that these are siblings is far less, unless inbreeding is favored. Given the lack of evidence of assortative/disassortative mating, this finding suggests that there is a clear propensity in the dark morph to mate with relatives. In general, however, inbreeding is assumed to lessen self and offspring fitness (i.e., inbreeding depression), mainly due to the accumulation of deleterious recessive alleles (Edmands 2007). These two incestuous pairs bred in 2013 and 2014, although only the pair of dark homozygous siblings successfully produced a chick in 2013; no chick fledged from either pair, or from any other pair in the colony, in 2014.

Different social and ecological constraints may enforce frequent interbreeding between relatives. For example, attraction to the natal nest-site may be a key factor favoring the occurrence of inbreeding if juveniles of both sexes experience similar attraction to natal colonies, as seems to be the case in this highly philopatric species (Walter 1979; Ristow et al. 1991; Gangoso et al. 2013). However, natal dispersal distances within the colony were higher in females than in males, a difference that would not seem to be great enough to counteract the likelihood of incestuous mating, provided that individuals have the ability to recognize relatives. In an experiment with the closely related American kestrel (*Falco sparverius*), captive females were given the opportunity to choose between two males differing in terms of their relatedness and nesting experience and, apparently, sexual display but not relatedness was used as a criterion in mate choice (Duncan and Bird 1989). An alternative explanation is that dark females may be less eligible as mates than pale ones, which may perhaps render inbreeding as the only option. However, our data show no evidence that males of any morph prefer pale females.

In Eleonora’s falcon, coloration seems to affect individuals’ performance and fitness in different ways. For instance, nestlings of different coloration have different immune and antioxidant capacities (Galván et al. 2010; Gangoso et al. 2011, 2015a). In addition, adults of the dark morph have higher blood parasite prevalence than pale ones (Gangoso et al. 2016). Moreover, males of alternative color morphs adopt different breeding tactics (Gangoso et al. 2015b) and mate choice may thus not be completely selectively neutral. We found that breeding success was both morph- and context-dependent. Our results showed that, whereas clutch size was similar in differently colored pairs, differences occur later on in the course of reproduction. In some years, dark males raised more offspring than pale males, regardless of female color morph. This result, which was significant in 2 of the study years, might point to the role of male quality and hunting abilities. In a recent study, Tate et al. (2016) showed that prey provisioning rates differed between color morphs of the black sparrowhawk (*Accipiter melanoleucus*) depending on light conditions: dark sparrowhawks delivered more prey in lower light conditions and white ones more prey in brighter conditions. These authors suggest that morph-specific crypsis promotes the maintenance of color variation in this species via disruptive selection along an environmental gradient. Despite this difference in prey provisioning rates, neither morph in isolation had a specific advantage in terms of productivity. However, a combination of morphs in adult pairs did affect productivity, with heterotypic pairs producing more—but of lower condition—offspring than homotypic ones (Tate et al. 2017). Although Eleonora’s falcons of alternative color morphs coexist sympatrically, we cannot rule out the possibility of morph-specific hunting strategies and hunting success.

The potential reproductive advantage for the dark—rarer—morph was not evident every breeding season as in some years no differences occurred or the productivity of pale males was higher, although not significantly (Figure 1). If dark males truly achieved higher reproductive output in a consistent fashion, one would expect an increase in the frequency of the dark morph in the population, unless this higher productivity is compensated by any disadvantage in relation to the pale morph at other stages of the life cycle. In our study population, however, dark males do not always perform better and genotype frequencies are stable and in equilibrium (Gangoso et al. 2015a). In an experimental study with color polymorphic guppies (*Poecilia reticulata*), Hughes et al. (2013) found strong evidence for rare-male advantage, possibly mediated by female mate preference. Nonetheless, these authors argue that detecting negative frequency-dependent selection under natural conditions is difficult, largely because the equilibrium frequencies of different morphs under this type of selection are those at which fitnesses are equal (Hughes et al. 2013). In Eleonora’s falcon, mating interactions may in turn interact with other factors such as the external abiotic conditions that drive food availability. This interaction could lead to the observed pattern of higher breeding output of dark males under certain environmental conditions but not necessarily under phenotype frequencies that differ from their equilibrium values. For instance, males of different morph adopt alternative breeding strategies, with pale males being highly colonial and dark males behaving more territorial (Gangoso et al. 2015b). This feature could have important implications for explaining the differences in breeding output between color morphs under certain environmental conditions. During prolonged periods of food scarcity associated with bad weather conditions (Viana et al. 2016), intraspecific predation becomes one of the most important factors affecting breeding output. Cannibalism usually occurs between nearby nests and is maximized in dense areas, mostly occupied by pale falcons (Gangoso et al. 2015b). In general, the years in which significant differences in productivity between male morphs occurred were also years when availability of food and, hence, mean productivity of the falcon population was low (see Figure 1). By living in less crowded neighborhoods, dark males may avoid high rates of intraspecific predation, especially when food is scarce, which may ultimately lead to a higher breeding output. Although color polymorphism in the Eleonora’s falcon is under strong genetic control (Gangoso et al. 2011), the possibility that these alternative breeding strategies have a genetic basis and are heritable is unknown. The long-term maintenance of polymorphism does not imply that alternative morphs achieve equal fitness, even if fitness depends on some aspects of the environment (Byers 2005; Sirkiä et al. 2010), as seems to be the case of our study population. For example, if one morph has a higher average fitness than the other, the maintenance of polymorphism in the long term may occur under the effect of mechanisms, such as negative frequency-dependent selection, that prevents the fixation of the more abundant morph (Gross 1996; Gigord et al. 2001). However, the fitness functions of each morph depending on their relative frequencies may not even cross at some specific point (Taborsky et al. 2008). For instance, the average reproductive success of one morph may always be higher than the other, irrespective of their relative frequency and thus, the relative abundance of morphs in the population will not be determined by frequency-dependent selection, but by other factors, such as, for instance, phenotypic quality (see Taborsky et al. 2008 and references therein). To fully understand the evolutionary processes that maintain color polymorphism in the Eleonora’s falcon, we need to address how social interactions, morph frequencies, phenotypic quality, and fitness parameters relate to one another and interact with the changing environmental conditions experienced by the birds in their breeding areas, on migration, and in their wintering areas in Madagascar.

## FUNDING

This work was partially supported by the Cabildo Insular de Lanzarote. During the writing of this manuscript, L.G. was supported by a Marie Sklodowska-Curie Fellowship of the European Commission (grant number: 747729 “EcoEvoClim”).

## Supplementary Material

arz013_suppl_Supplementary_material_1

arz013_suppl_Supplementary_material_2
