# Supplementary material for: Breeding success but not mate choice is phenotype- and context-dependent in a color polymorphic raptor
Source: Behav Ecol. 2019 Feb 9;30(3):763–9. doi: 10.1093/beheco/arz013 (PMC6562304; doi:10.1093/beheco/arz013)
Supplement: arz013_suppl_Supplementary_material_1 [file arz013_suppl_supplementary_material_1.pdf]

Appendix 1

Supplementary methods

Breeding success but not mate choice is phenotype- and context-dependent in a color polymorphic raptor

Laura Gangoso and Jordi Figuerola

Table S1. Number and distribution of pair types within the subset of individuals followed from birth to recruitment in the breeding population. The first row shows the sex and morph (P=pale and D=dark) of the target individual as well as the color morph of its biological (Bfather and Bmother) and foster (Ffather and Fmother) parents. The column "pair" denotes the color morph of the mate and "nestID" corresponds to the nest site where the individual was recruited (identical numbers indicate that the nest is the same for two individuals within a pair).

sex	morph	Bfather	Bmother	Ffather	Fmother	pair	nestID
M	P	P	P	P	P	P	1
F	P	P	P	P	P	P	2
M	P	P	P	P	P	P	2
F	P	P	D	P	D	P	3
M	P	D	P	D	P	P	3
M	D	P	D	P	D	P	4
F	P	P	P	P	P	D	4
F	P	D	P	D	P	P	5
F	P	P	P	P	P	P	6
F	P	P	P	P	P	P	7
M	P	D	D	P	P	P	8
F	P	P	P	P	P	P	8
F	P	P	P	P	P	P	9
F	D	P	D	D	P	P	10
M	D	D	P	D	D	P	11
M	P	P	P	P	P	P	12
F	P	P	D	P	D	P	12
F	P	P	P	P	P	D	13
F	P	P	D	P	P	P	14
F	P	P	P	P	P	P	15
F	D	D	D	D	D	D	16
M	D	D	D	D	D	D	16
F	P	P	P	P	P	D	17
F	P	P	P	P	P	P	18
F	P	P	D	P	D	P	19
M	D	P	D	D	P	P	20
F	P	P	P	P	P	D	20
F	P	P	P	P	P	P	21

M	P	P	P	P	P	P	22
M	P	P	P	P	P	P	23
F	P	P	P	P	P	P	24
M	P	P	P	P	P	P	24
F	P	P	D	P	D	P	25
M	P	P	P	P	P	P	26
F	P	D	P	P	P	P	26
F	P	P	P	P	P	P	27
M	P	P	P	P	P	P	27
M	P	P	P	P	P	D	28
F	P	P	P	P	P	D	29
F	P	D	P	D	P	D	30
M	P	P	P	P	P	P	31
M	D	D	P	D	P	P	32
F	P	P	P	P	P	P	33
F	P	P	P	P	D	P	34
F	P	P	P	P	P	D	35
F	P	P	D	P	D	P	36
M	D	D	P	D	P	P	37
F	P	P	P	P	P	D	37
M	P	D	P	P	D	P	38
M	P	P	P	P	P	D	39
F	D	D	D	D	D	P	39
M	D	P	D	P	D	P	40
M	P	P	P	P	P	P	41
M	D	D	P	D	P	D	42
F	D	D	P	D	P	D	42
M	P	P	P	P	P	P	43
M	P	P	P	P	P	P	44
M	P	P	P	P	P	P	45
M	P	P	P	P	P	P	46
M	P	P	P	P	P	D	47
F	D	P	D	P	D	P	48
M	P	P	P	P	P	P	49
F	P	P	P	P	P	P	50
M	D	D	P	D	P	P	51
M	P	P	P	P	P	D	52
F	P	P	P	P	P	P	53
M	P	P	P	P	P	P	54
F	P	P	P	P	P	D	55
M	P	P	P	P	P	P	56
M	P	P	D	P	D	P	57
F	P	P	D	P	D	P	58
M	P	P	P	P	P	P	58
M	P	P	P	P	P	D	59
M	P	P	D	P	D	D	60
M	D	D	P	D	P	P	61
M	P	P	P	P	P	D	62

M	P	P	P	P	P	P	63
M	P	P	P	P	D	P	64
M	D	P	P	P	D	P	65
M	D	D	P	D	P	P	66
M	P	P	P	P	P	P	67
F	P	D	P	P	P	P	68
F	P	D	P	D	P	P	69
M	P	P	P	P	P	P	69
F	P	P	P	P	P	P	70
M	P	P	P	P	P	D	71
F	P	P	D	P	D	D	72
M	P	P	P	P	P	P	73
F	P	D	D	D	D	P	74
M	P	P	P	P	P	P	75
F	P	D	P	D	P	P	76
M	D	P	D	P	D	P	77
F	P	P	P	P	P	D	77
M	P	D	D	D	D	P	78
F	P	P	P	P	P	P	78
F	P	D	P	D	P	P	79
M	P	P	P	P	P	P	80
M	P	P	P	P	P	P	81
F	P	P	P	P	P	P	81
F	P	D	D	D	D	D	82
M	D	D	D	D	D	P	83
F	P	P	D	P	D	D	84
M	P	P	P	P	P	P	85
M	P	P	P	P	P	P	86
F	P	P	P	P	P	P	86
F	P	P	P	P	P	P	87
F	P	P	P	P	P	P	88
M	P	P	P	P	P	P	89
F	P	D	P	D	P	P	89
