# Supplementary material for: Breeding success but not mate choice is phenotype- and context-dependent in a color polymorphic raptor
Source: Behav Ecol. 2019 Feb 9;30(3):763–9. doi: 10.1093/beheco/arz013 (PMC6562304; doi:10.1093/beheco/arz013)

Appendix 2

Supplementary results

Breeding success but not mate choice is phenotype- and context-dependent in a color polymorphic raptor

Laura Gangoso and Jordi Figuerola

Table S2. Results of logistic regression model assessing patterns of mating within the subset of recruited individuals (N=109). The first two tables show the results of the model and the comparison of models based on the Likelihood Ratio Test after single term deletions for the subset of initially chosen individuals (N= 87). The third and forth tables show the results of the same model but using the subset of previously discarded individuals (N=87).

Initially chosen individuals				
Variable	Estimate	Std. error	Z value	Pr(>|z|)
Intercept	-1.1568	0.4511	-2.564	0.0103
MorphD	-0.9769	1.0598	-0.922	0.3566
SexM	-0.1719	0.5723	-0.300	0.7638
Biol Father D	-0.5629	1.2246	-0.422	0.6732
Biol Mother D	0.1377	0.9896	0.139	0.8893
Foster Father D	0.8232	1.4955	0.550	0.5820
Foster Mother D	0.1063	0.9678	0.110	0.9125

Variable	Df	Deviance	AIC	LRT	Pr(>Chi)
<none>		89.732	103.73		
Morph	1	90.639	102.64	0.90689	0.3309
Sex	1	89.822	101.82	0.09031	0.7638
Biol Father	1	89.923	101.92	0.19115	0.6620
Biol Mother	1	89.751	101.75	0.01925	0.8896
Foster Father	1	90.054	102.05	0.32208	0.5704
Foster Mother	1	89.744	101.74	0.01206	0.9125

Previously discarded individuals				
Variable	Estimate	Std. error	Z value	Pr(>|z|)
Intercept	-0.7931	0.3953	-2.006	0.0449
MorphD	-0.7022	1.0425	-0.674	0.5006
SexM	-0.7007	0.5587	-1.254	0.2098
Biol Father D	-0.8659	1.4831	-0.584	0.5593
Biol Mother D	0.2651	1.1479	0.231	0.8174
Foster Father D	0.8484	1.6198	0.524	0.6005
Foster Mother D	-0.0049	1.0901	-0.005	0.9963

Variable	Df	Deviance	AIC	LRT	Pr(>Chi)
<none>		92.637	106.64		
Morph	1	93.113	105.11	0.47694	0.4898
Sex	1	94.251	106.25	1.61442	0.2039
Biol Father	1	93.016	105.02	0.37925	0.5380
Biol Mother	1	92.690	104.69	0.05389	0.8164
Foster Father	1	92.931	104.93	0.29458	0.5873
Foster Mother	1	92.637	104.64	0.00002	0.9963

Table S3. Results of the cumulative link mixed models (CLMM) assessing differences in clutch size between differently colored individuals (N= 740 breeding attempts) and comparison of models based on the Likelihood Ratio Test after single term deletions.

Variable	Estimate	Std. error	Z value	Pr(>|z|)
Male D	-0.01289	0.19879	-0.065	0.948
Female D	-0.14776	0.21724	-0.689	0.496
Male D: Female D	0.04266	0.50189	0.085	0.932

	Df	AIC	LRT	Pr(>Chi)
<none>		1352.4		
Male	1	1348.5	0.00114	0.973
Female	1	1349.0	0.50792	0.476
Male:Female	1	1350.5	0.00723	0.9322

Figure 1A. Box plot showing the fitted probabilities of the model for dark and pale males with the year effects at their conditional modes. The line within boxes indicates the median, the edges of the boxes the first (Q1) and third (Q3) quartiles, and the whiskers extend 1.5 times the interquartile range.

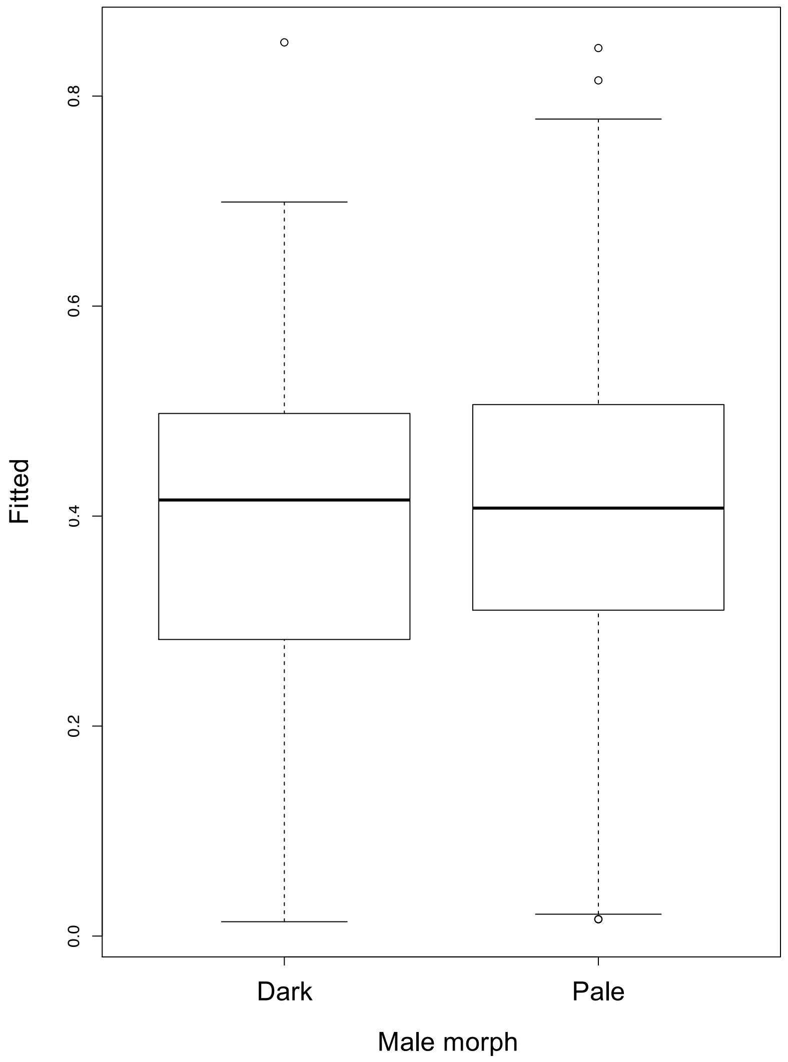

Supplement: arz013_suppl_Supplementary_material_2 [file arz013_suppl_supplementary_material_2.pdf]
